# Sick leave after arthroscopic meniscus repair vs. arthroscopic partial meniscectomy

**DOI:** 10.1016/j.ocarto.2023.100340

**Published:** 2023-01-20

**Authors:** Fredrik Boric-Persson, Aleksandra Turkiewicz, Paul Neuman, Martin Englund

**Affiliations:** a Lund University, Skåne University Hospital, Department of Orthopaedics, Malmoe, Sweden; b Clinical Epidemiology Unit, Orthopaedics, Department of Clinical Sciences Lund, Faculty of Medicine, Lund University, Lund, Sweden

**Keywords:** Meniscus, Meniscus repair, Arthroscopy, Sick leave, Partial meniscectomy

## Abstract

**Objective:**

To evaluate sick leave after meniscal repair vs arthroscopic partial meniscectomy (APM) and, for comparison, vs the general population.

**Method:**

Using Swedish register data we included all employed persons aged 19–49 years in the general population of Skåne region and identified those having had meniscus repair or APM in the period of 2005–2012. We retrieved data on sick leave during 1 year before until 2 years after surgery. We used logistic regression to estimate the risk differences of being on sick leave and negative binomial model to analyze differences in the number of days on sick leave.

**Results:**

We included 192 persons with meniscus repair, 2481 with APM, and 376 ​345 references without meniscus surgery. Of these, 55% of meniscus repair group, 43% of APM group had any sick leave in the 2-year period following the surgery, while 17% of the references were on sick leave in the corresponding period. The mean (SD) number of days of sick leave after meniscus repair was 55 (77) days and for APM 37 (86) days. Meniscus repair was associated with higher probability of sick leave compared to APM with an adjusted risk difference of 0.13 (95% CI 0.07–0.19).

**Conclusion:**

Persons undergoing meniscus repair have more frequent and 37% longer periods of sick leave in the short term than persons undergoing APM. However, sick leave in the long-term warrant further attention as successful repair may be associated with less knee osteoarthritis development than APM.

## 1 Introduction

Acute meniscus tear is a common knee injury, with an incidence around 70/100 ​000 [1]. During most of the later part of 20th century arthroscopic partial meniscectomy (APM) has been the treatment of choice for meniscus tears, but during the 21st century meniscus repair has become increasingly frequent when repair is possible [[2], [3], [4]]. It is generally considered that suture of the meniscus, with added postoperative ambulatory restrictions, results in longer absence from work than APM. Studies confirming this notion, however, are lacking. There are only a few smaller, single center studies dealing with return to sports in athletes after meniscus repair [[5], [6], [7]]. It has been estimated that meniscus repair surgery is more expensive than APM, taking into consideration the longer time in the operating room, longer rehab and cost of implants, even without adding the costs of prolonged work absence [8]. There is evidence that APM hastens progression of osteoarthritis [9,10], and meniscus repair has been reported to be more cost effective than APM, when focusing on the anticipated decreased risk of osteoarthritis development, increased quality of life and potentially less need for total knee arthroplasty [11].

There are a few studies describing sick leave after APM [[12], [13], [14], [15]], but no direct comparisons with meniscus repair. Thus, our aim was to compare sick leave occurrence and duration in young and middle-aged patients undergoing APM or meniscal repair and relate these to sick leave in the general population in southern Sweden, which has a publicly financed social security system.

## 2 Patients and methods

### 2.1 Ethics

The study was approved by the Ethics Committee (ethical approval IRBnumber: IRB_2011-432) and adheres to the rules of the Helsinki Declaration of 1975, as revised in 2000.

### 2.2 Register resources

#### 2.2.1 Skåne healthcare register (SHR)

Within public healthcare in the Skåne region (population size 1 ​288 ​908, in the year 2014) all surgeries are recorded in the SHR. Private clinics do not report to the SHR, however, they did not perform meniscus repairs during this time period. From the register we retrieved surgical date, surgical type (Swedish version of NOMESCO Classification of Surgical Procedures; KKÅ97) and diagnostic codes registered according to the International Classification of Diseases, version 10 system [16]. The positive predictive value (PPV) of a surgical procedure code or an ICD10-code being correct in the register overall is 85–95%, with PPV for knee trauma and knee surgery being above 90% (90–92%) [17].

#### 2.2.2 The population register

In Sweden all legal residents are included in the population register which is used for a variety of purposes by authorities and private corporations including tax agency, health care, banks etc. It is continuously updated with information on current residential address and deaths. From the register, we retrieved information on age, sex and residential area.

#### 2.2.3 Swedish social security agency database (SSIA)

For employed persons, data is registered for all periods of sick leave that lasts longer than 14 days and that is reimbursed by the SSIA. The available data include personal identification number, dates of start and end of a sick leave period and extent of sick leave (25, 50 75 or 100%). Inhabitants in Sweden who cannot work, either because of illness or injury are entitled to sickness benefits (compensation for reduced income) counting from day 2 of a reported sickness period. For individuals with employment, day 2–14 is paid for by their employer. If the period of illness exceeds 14 days, it is thereafter reimbursed by the Swedish Social Insurance Agency (SSIA). If an individual is unemployed, on parental leave or a student, then SSIA registers and reimburses them from day 2 if they apply for the benefit. A smaller group of self-employed is reimbursed according to different rules. Around 76% of all the people in the SSIA register pertains to the group “employed”, with the most homogenous data. Thus, in this study we only included employed persons and could only capture sick leave episodes with duration of at least 14 days.

#### 2.2.4 The longitudinal database (LISA) of statistics Sweden

From LISA database we retrieved data on employment status, marital status (married or registered partner or not), income, highest education level reached, if born in Sweden and occupation as classified according to Swedish version of ISCO 88 (SSYK 96 (Standard för Svensk Yrkesklassificering 1996)) Using this we categorized the different occupations into 3 groups, “Light knee load” mainly office work (occupational groups: 1–222, 224–244, 246–322, 324–346, 348–499), “Medium knee load”, defined as light manual labor (occupational groups: 223, 323, 500–514, 516–599, 900–920, 922–930, 932–999) and finally “High knee load” containing work such as construction workers, firefighters etc. (occupational groups: 245, 347, 515, 600–899, 921, 931, 0).

### 2.3 Inclusion and exclusion criteria

**Fig. 1:**
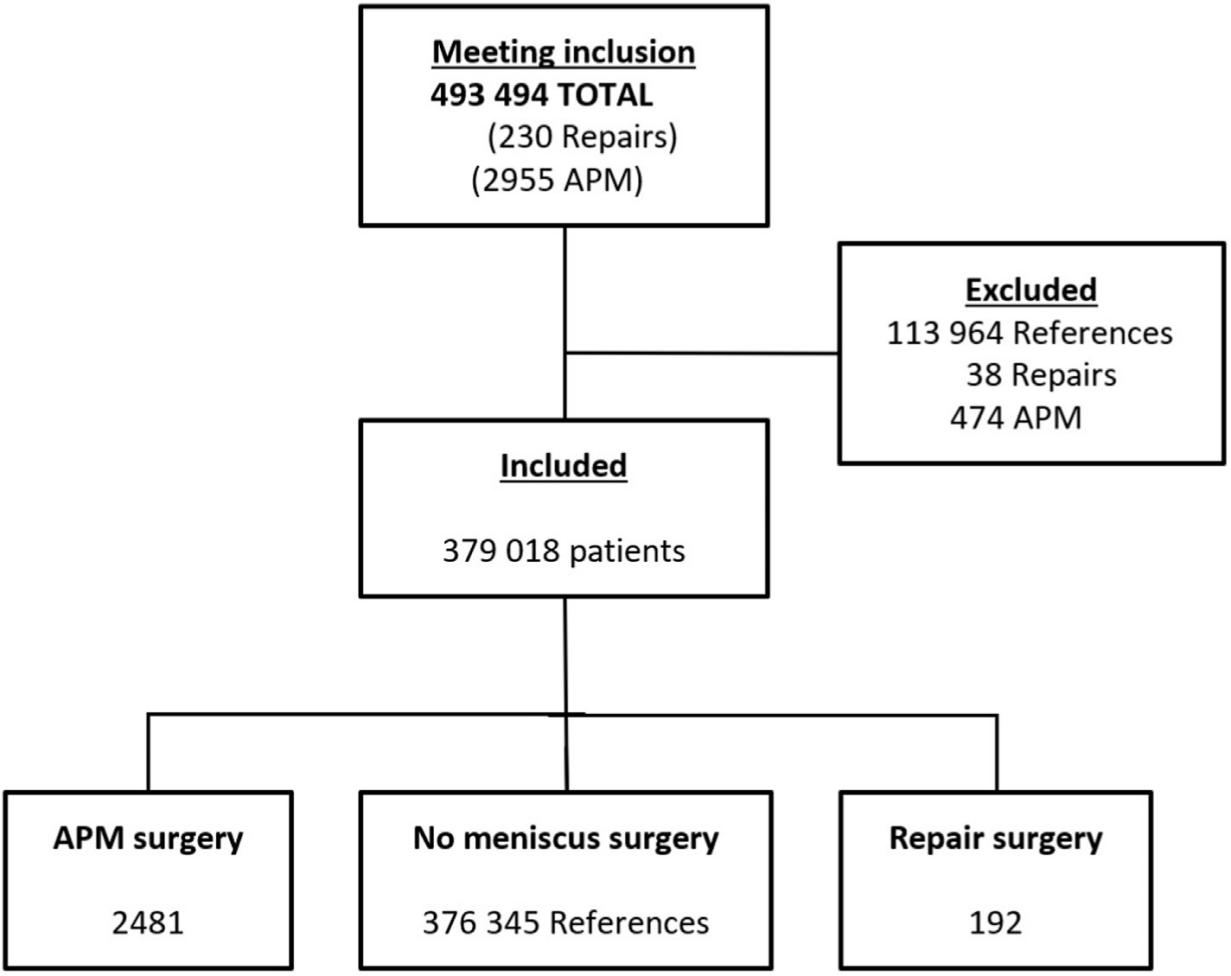
Flowchart of study inclusion and exclusion process.

We identified all male and female patients in the SHR having had APM (code NGD11) or meniscus suture (NGD21) with an ICD-10 diagnosis of meniscus injury (S83.2 or M23.2) during January 1, 2005 until December 31, 2012 in age interval 19–49 years old at the time of surgery, and registered resident in Skåne at the end of the year of surgery and one calendar year *before* the surgery date. As control group we identified all persons not having meniscal surgery (any NGD code), but having had at least one healthcare visit during January 1, 2005 until December 31, 2012, age interval 19–49 years old at the time of the visit, and registered resident in Skåne at the end of the year of inclusion and one calendar year before the year of the visit. For each person we randomly sampled one visit as the index date. We excluded a person, from all groups, if during 4 years preceding the inclusion date they were diagnosed with any of the following: fracture in the knee (ICD-10 code: S82.1, S82.9, S72.4 and 72.9), dislocation of knee (ICD-10 code: S83.1), rupture of Medial Collateral Ligament (MCL)/Lateral Collateral Ligament (LCL) (ICD-10 code: S83.4), diagnosis of knee osteoarthritis (ICD-10 code: M17.1-9), other meniscus surgery at index date (NGD20 or NGD22) previous meniscus surgery (surgery codes: NGD11-99, NGD21-99). Further, we excluded persons with disability pension (at the surgery/index date), sick leave in any form lasting the whole 360-day period before surgery/index date, since any prolonged sick leave on their part would probably not be attributed to the performed meniscus surgery. We excluded all patients with surgeries to the meniscus within 4 years prior, since we didn't have laterality, and this was a way to exclude re-operations to the same knee [18]. We also excluded all patients with diagnosed knee osteoarthritis, since they would have a very low probability of getting a meniscus repair which would make the groups less comparable. In Table 4 and Fig. 1 a summary of the inclusion and exclusion process is presented.

**Table 1:** Baseline characteristics of the patients in the two surgical groups and in the reference population, at index date.

	APM	Repair	General Population
N	2481	192	376 ​345
Women, n (%)	627 (25)	71 (37)	196 ​701 (52)
Age at inclusion mean (SD) years	35 (9)	27 (7)	34 (10)
Income, mean (SD) 100.000 SEK	2.28 (1.6)	1.76 (0.9)	2.02 (1.9)
Marital status, n (%)	1118 (45)	43 (22)	154 ​889 (41)
Born abroad, n (%)	350 (14)	34 (18)	69 ​787 (19)
Educational level, n (%)			
0–9 years	232 (9)	23 (9)	35 ​672 (9)
10–12 years	1460 (59)	103 (41)	196 ​389 (52)
13–14 years	345 (14)	30 (12)	57 ​772 (15)
>15 years	440 (18)	36 (14)	85 ​209 (23)
Occupational group, n (%)			
1 ​= ​light knee load	955 (38)	60 (31)	145 ​762 (39)
2 ​= ​medium knee load	534 (22)	48 (25)	111 ​698 (30)
3 ​= ​high knee load	823 (33)	47 (24)	73 ​829 (20)
9 ​= ​missing data	169 (7)	37 (19)	45 ​056 (12)
Other procedures at time of surgery, n (%)			
ACL reconstruction	238 (10)	52 (27)	55 (0)
Cartilage surgery	154 (6)	11 (6)	44 (0)
Meniscus diagnosis at time of surgery, n(%)			
Acute meniscus tear (S83.2)^a^	103 (4)	27 (14)	13 (0)
Derangement of meniscus, old tear (M23.2)	2391 (96)	169 (88)	483 (1)
ACL diagnosis at time of surgery, n (%)			
Acute ACL rupture (S83.5)	126 (5)	20 (10)	152 (0)
Chronic Instability (M23.5)	546 (22)	78 (41)	433 (0)

a ICD-10 diagnosis codes within ().

**Table 2:** The number of sick days specified in each group.

		Total, n	Men, n (%)	Women, n (%)	ACL rupture, n (%)	Mean age, years	People, n (%) with any sick leave	Mean number of days with sick leave	
								Mean # days of those on sick leave	Mean # days in all patients
Meniscus Repair									
	No ACL-surgery	140	88 (63)	52 (37)	46 (33)	27	77 (55)	96	53
	No ACL-surgery, ACL intact	94	59 (63)	35 (37)	N/A	27	51 (54)	91	49
	No ACL-surgery, ACL rupture	46	29 (63)	17 (37)	N/A	28	26 (57)	107	60
	ACL surgery	52	33 (64)	19 (36)	52 (100)	26	27 (52)	114	60
	**All**	**192**	**121 (63)**	**71 (37)**	**98 (51)**	**27**	**105 (55)**	**101**	**55**
APM									
	No ACL-surgery	2243	1680 (75)	563 (25)	435 (19)	36	910 (41)	82	33
	No ACL-surgery, ACL intact	1808	1359 (75)	449 (25)	N/A	36	691 (38)	77	29
	No ACL-surgery, ACL rupture	435	321 (74)	114 (26)	N/A	33	219 (50)	98	49
	ACL surgery	238	174 (73)	64 (27)	237 (99)	29	159 (67)	99	66
	**All**	**2481**	**1854 (75)**	**627 (25)**	**672 (27)**	**35**	**1069 (43)**	**85**	**37**
References									
	No ACL-surgery	376 ​290	179 ​609 (48)	196 ​681 (52)	N/A	33	63 ​692 (17)	104	18
	ACL surgery	55	35 (64)	20 (36)	N/A	28	33 (60)	111	67
	**All**	**376 ​345**	**179 ​644 (48)**	**196 ​701 (52)**	**585 (0.2)**	**33**	**63 ​725 (17)**	**104**	**18**

APM ​= ​arthroscopic partial meniscectomy; N/A ​= ​non applicable.

**Table 3:** Adjusted risk difference and risk ratio of being on sick leave after meniscus repair versus APM and ratio of mean days on sick leave for persons with sick leave.

Model	Risk difference (95% CI)	Risk ratio (95% CI)	Ratio of mean days on sick leave (95% CI)
Adjusted for age, sex, income category, if born abroad, ​occupational group	0.15 (0.08, 0.22)	1.8 (1.5, 2.2)	1.37 (1.15,1.64)
Additionally adjusted for ACL rupture diagnosis	0.12 (0.05, 0.19)	1.3 (1.1,1.5)	1.25 (1.06, 1.49)
Additionally adjusted for concomitant ACL reconstruction	0.13 (0.05, 0.20)	1.3, (1.1, 1.5)	1.25 (1.05, 1.48)

**Table 4:** Inclusion and exclusion criteria.

INCLUSION
- All patients in the SHR between 2005 and 01-01 and 2012-12-31
- Index date defined as date of surgery or healthcare visit for references
- Age 19–49 years
- Meniscus injury (ICD10: S.832 or M.232) and meniscus surgery (NGD11 or NGD21)
- For reference group, NOT having had meniscus surgery (any NGD surgical code)
**EXCLUSION**
- Long term sick leave (more than 364 days)
- Not residing in the Skane region
- Having other meniscus surgery (NGD20 or NGD22) at index date
- Receiving disability pension
- Any of the following diagnosis during 4 years prior to index date:
1. Fracture (ICD-10: S82.1, S82.9, S72.4 and 72.9)
2. Dislocation of knee (ICD-10: S83.1)
3. Ligament rupture (MCL/LCL) (ICD-10: S83.4)
4. Knee osteoarthritis (ICD-10: M17.1-9)
5. Previous meniscus surgery (NGD11-99, NGD21-99)

### 2.4 Surgical method

During the study period, all hospitals in the region for most cases used the same 2 portal arthroscopy technique (one work portal) and all-inside suture technique, except for some anterior horn meniscal repairs, where outside-in technique was employed. Patient were under general anesthesia. Whether a tourniquet was used or not during surgery was not recorded. However, previous research suggest that this does not significantly alter the length of return to work [19].

### 2.5 Postoperative regime

Standard postoperative care in southern Sweden after most common meniscus repair surgery includes walking with 2 crutches with full weightbearing during 6 weeks to normalize gait. Patients are prohibited to put wight to the knee with more flexion than 90° for 6 weeks post surgery. They are encouraged to neuromuscular rehabilitation of the knee under supervision by a physiotherapist until normal knee function is restored. All patients with high knee loading occupations are prescribed sick leave for up to 6 months to avoid putting the meniscal repair at risk to re-rupture. The same timeframe of 6 months is also the recommendation to avoid sports activities, especially with high knee loads in flexion and rotation.

After APM, standard postoperative care in Sweden is full weightbearing, 2 crutches for walking to normalize gait in some cases when needed, as well as rehabilitation of the knee supervised by a physiotherapist until normal knee function is restored. The rehabilitation and sick leave prescribed after APM surgery is governed by knee symptoms, and with work demanding high knee function, usually for 2–4 months.

### 2.6 Outcome definition and follow-up time

Our outcomes included a binary variable denoting if a person was on any sick leave longer than 14 days during the first two years after the surgery. Then, we also calculated number of net days on any sick leave during the initial 2 years after the surgery. Every day that a person received any amount of sick leave compensation (partial or full day), was counted as ‘1’ day of sick leave.

### 2.7 Statistical analysis

We used logistic regression and the method of standardization to compute the risk ratio and risk differences of being on sick leave. Then, only among persons on sick leave, we fitted a negative binomial model with robust standard errors to estimate the ratio of mean number of days on sick leave between the groups. Both regression models were adjusted for age, sex, marital status, level of education, if born outside Sweden, and income. Further, as rupture of anterior crucial ligament is common in conjunction with a traumatic meniscal tear and associated with more serious surgery, and thus potentially longer sick leave, we adjusted the models for the presence of ACL surgery (codes NGE41) at the time of the surgery.

All statistical analyses were performed using IBM SPSS version 26, New Orchard Road, Armonk, New York, United States and Stata (StataCorp. 2021. *Stata Statistical Software: Release 17*. College Station, TX: StataCorp LLC.).

## 3 Results

Out of the general population of Skåne region of 1.3 million inhabitants, we identified 493 ​494 subjects fulfilling the inclusion criteria. A total of 379 ​018 subjects were included after applying our exclusion criteria (192 patients with meniscus repair, 2481 with APM and 376 ​345 reference subjects). (Table 1). During the two years after the surgery date, 55% in the repair group and 43% in the APM group had had any episode of sick leave longer than 14 days. Numbers for each subgroup is presented (Table 2).

After adjusting for potential confounders, the risk ratio of being on sick leave for more than 14 days during the two years after surgery in patients undergoing meniscus repair compared to the general population was 3.6 (95% CI 3.3., 4.0), with corresponding risk difference of 0.45 (95% CI 0.38, 0.51). The corresponding estimates for APM was 2.6 (95% CI 2.5 to 2.7), and 0.27 (95% CI 0.25, 0.29), respectively. The adjusted comparison of meniscus repair with APM yielded a risk ratio of 1.8 (95% CI 1.4 to 2.1) with corresponding risk difference of 0.13 (95% CI 0.07, 0.19).

Among those on sick leave, the sick leave was on average 37% (95% CI 15%, 64%) longer after meniscus repair than APM.

We further adjusted for the presence of ACL rupture with or without concomitant ACL reconstruction in calculating the risk of being on sick leave for meniscus repair versus APM. The results were similar (Table 3).

To investigate if there was any sex-difference we calculated the adjusted risk difference for being on sick leave for meniscus repair versus APM to be 0.15 (95% CI 0.07, 0.22) for men and 0.10 (95% CI 0.00, 0.20) for women.

**Fig. 2:**
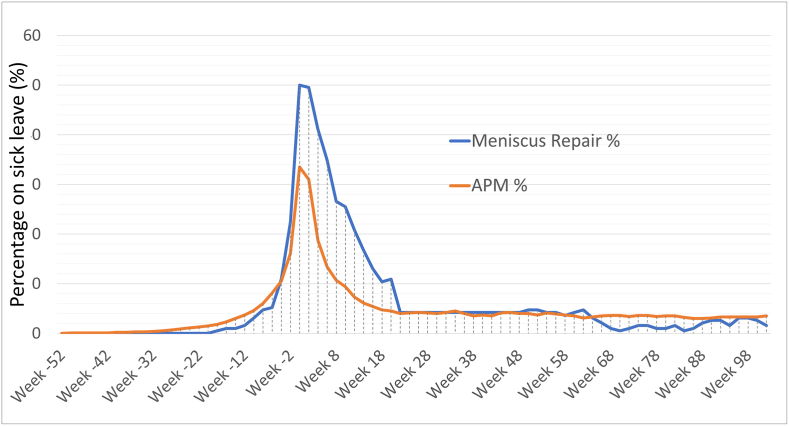
Percentage of patients on sick leave per week from 1 year before to 2 years after surgery, all patients.

**Fig. 3:**
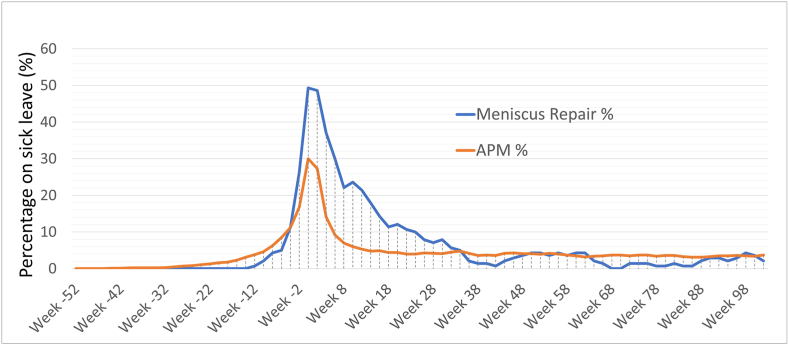
Percentage of patients with only meniscus surgery (without any ACL-surgery) on sick leave per week from 1 year before to 2 years after surgery.

Table 3: We illustrate the crude pattern (unadjusted for age and sex differences) of sick leave over time, both total numbers (Fig. 2) and after excluding ACL-reconstructions (Fig. 3). The meniscus repair-group had a higher percentage and longer duration of sick leave after surgery, but then over time return to a somewhat lower proportion on sick leave than the APM group.

The curve for sick leave after APM flattens out after the postoperative phase, but never returned to the preoperative levels, instead between 4 and 5% of patients remained on sick leave both 1 and 2 years postoperatively.

## 4 Discussion

We investigated the frequency and average length of sick leave longer than 14 days after meniscus repair and APM in southern Sweden during a 10-year time frame. Our findings reveal that both male and female patients after meniscus repair are on sick leave to a higher extent, and for longer periods of time than after APM. However, we found no clinically relevant sex differences in the propensity to be on sick leave after repair. Interestingly, this differs from the general pattern of women being on more and longer sick leave, both in the general population and after APM [15]. There was a minor difference in the number of patients working with high knee demand jobs between the APM and repair group. As expected, there were slightly more APMs in the group with high knee demands, since choosing an APM over a repair could be mainly a request from the patient after informed consent regarding meniscal surgical options and consequences. It could also be a result of this category having more complex degenerative tears not suitable for repair surgery.

The goals of meniscus surgery for a traumatic tear is to alleviate symptoms while still saving as much of the meniscus function as possible. It is well established that APM is associated with an increased risk of osteoarthritis, but also that meniscus suture repair has a higher risk of reoperations than APM. The decision of whether to perform meniscus surgery with repair, APM or with non-operative management, depends on several factors such as meniscal quality and tear type, concomitant knee injuries and osteoarthritis, occupation and knee-joint loading, as well as the individual skill and preferences of the surgeon.

Unfortunately we lack the specific reasons for the sick leave due to data not available in the register. However, we capture “all cause” reimbursed sick leave longer than 14 days in an entire population based on prospectively ascertained data, not patients’ self reports. The agreement between retrospective self-reporting and high quality prospectively ascertained register data on sick leave duration has been found to be poor [20]. The data also show all-cause sick leave for 2 years following surgery, to better include complications and reoperations, not only the initial injury and surgery. Further we have contrasted the extent of sick leave to the corresponding extent of sick leave in the background population, which is on average 9 days per year (range 5–12 days) during this period. While our aim was to compare sick leave associated with two different surgical procedures for traumatic meniscal tears, and the diagnosis of meniscus tear was confirmed by arthroscopy, we were concerned of not having a similarly high validity of the coding in patients not having surgery. Hence, we opted not to monitor a group of patients with a diagnosis of meniscal tear without having had knee surgery. Unfortunately, the SHR register does not cover information if the meniscal tear was, for instance, confirmed by MRI or if it was purely made on clinical examination in the patients without surgery. To get homogenous data, we included only people with an employment and the right to sick leave reimbursement from SSIA.

Previous studies have suggested that the mean duration of sick leave is 9–17 days for APM, and more than one third of patients had more than 14 days of sick leave (in median 31 days) [12,13,15]. In a study by Von Essen et al. authors reported that the mean number of sick days (counting from injury, not surgery) in a cohort of ACL-surgeries without meniscus injury, to be 88.5 days (SD 50.2) [21]. This is in line with our findings, that after meniscus repair with concurrent ACL reconstruction, the number of sick days is longer than without ACL reconstruction (114 days vs 96 days).

Being on sick leave after surgery, is not only a cost for society, but also there is a substantial reduction in the patients’ personal income [22]. Reducing the time spent away from work would be beneficial to both. This investigation of mapping the current sick leave after surgery could be the first step in this effort.

Finally, it would be interesting to compare the length of sick leave also with non-operatively treated meniscus tears. A recent RCT comparing meniscal surgery with exercise in this patient group has been published recently, and a similar dataset could be used for measuring sick leave as well as knee function [23]. Register-based research is otherwise difficult in non-operatively treated tears. They do not routinely visit the hospitals and are likely less captured with the correct diagnosis.

## 5 Conclusion

This study confirms the notion that meniscus repair without ACL reconstruction, is associated with more sick leave episodes longer than 14 days, than APM in the short term. However, the long-term consequences warrant further attention as successful repair may be associated with less knee osteoarthritis development [24], and thus less sick leave and societal costs later in life.

## Author contributions

Fredrik Boric-Persson (Fredrik.boric-persson@med.lu.se) was involved in study conception and design, the data analysis, interpretation of results, drafted the manuscript and final approval. Responsible for the integrity of the whole article. Aleksandra Turkiewicz was involved in study design, retrieval of data and statistical data analysis, interpretation of results, manuscript revision and final approval. Paul Neuman was involved in interpretation of data, manuscript revision and final approval. Martin Englund was involved in study conception and design, interpretation of results, manuscript revision and final approval.

## Role of the funding source

This study was funded by the 10.13039/501100004359Swedish Research Council, the 10.13039/501100006075Greta and Johan Kock Foundation, the 10.13039/501100007949Swedish Rheumatism Association, the Österlund Foundation, and Governmental Funding of Clinical Research within the National Health Service (ALF). The funding sources had no role in the design, collection, and interpretation of the data or the decision to submit for publication.

## Declaration of competing interest

No conflict of interests for any of the authors.

## References

[1] Nielsen A.B., Yde J. (1991). Epidemiology of acute knee injuries : a prospective hospital investigation. J. Trauma: Injury Infect. Critic. Care.

[2] Abram S., Price A.J., Judge A., Beard D.J. (2020). Anterior cruciate ligament (ACL) reconstruction and meniscal repair rates have both increased in the past 20 years in England: hospital statistics from 1997 to 2017. Br. J. Sports Med..

[3] Jacquet C., Pujol N., Pauly V., Beaufils P., Ollivier M. (2019). Analysis of the trends in arthroscopic menis- cectomy and meniscus repair procedures in France from 2005 to 2017. Orthop. Traumatol. Surg. Res..

[4] Abrams G.D., Frank R.M., Gupta A.K., Harris J.D., Mccormick F.M., Cole B.J. (2013). Trends in meniscus repair and meniscectomy in the United States, 2005-2011. Am. J. Sports Med..

[5] Logan M., Watts M., Owen J., Myers P. (2009). Meniscal repair in the elite athlete results of 45 repairs with a minimum 5-year follow-up. Am. J. Sports Med..

[6] Alvarez-Diaz P., Alentorn-Geli E., Llobet F., Granados N., Steinbacher G., Cugat R. (2016). Return to play after all-inside meniscal repair in competitive football players: a minimum 5-year follow-up. Sport Traumatol. Arthrosc..

[7] Willinger L., Herbst E., Diermeier T., Forkel P., Woertler K., Achtnich A. (2019). High short-term return to sports rate despite an ongoing healing process after acute meniscus repair in young athletes. Sport Traumatol. Arthrosc..

[8] Lester J.D., Gorbaty J.D., Odum S.M., Rogers M.E., Fleischli J.E. (2018). The cost-effectiveness of meniscal repair versus partial meniscectomy in the setting of anterior cruciate ligament reconstruction. Arthrosc.: J. Arthrosc. Relat. Surg..

[9] Santana D.C., Oak S.R., Jin Y., Rothy A., Lee L., Jones M.H. (2022). Increased joint space narrowing after arthroscopic partial menis- cectomy: data from the osteoarthritis initiative. Am. J. Sports Med..

[10] Collins J.E., Shrestha S., Losina E., Marx R.G., Guermazi A., Katz J.N. (2022). Five-year structural changes in the knee among patients with meniscal tear and osteoarthritis: data from a randomized controlled trial of arthroscopic partial meniscectomy versus physical therapy. Arthritis Rheumatol..

[11] Feeley B.T., Liu S., Garner A.M., Zhang A.L., Pietzsch J.B. (2016). The cost-effectiveness of meniscal repair versus partial meniscectomy: a model-based projection for the United States. Knee.

[12] Rockborn P., Hamberg P., Gillquist J. (2000). Arthroscopic meniscectomy: treatment costs and postoperative function in a historical perspective. Acta Orthop. Scand..

[13] Morrissey M.C., Milligan P., Goodwin P.C. (2006). Evaluating treatment effectiveness: benchmarks for rehabilitation after partial meniscectomy knee arthroscopy. Am. J. Phys. Med. Rehabil..

[14] Rongen J.J., Govers T.M., Buma P., Rovers M.M., Hannink G. (2017). Arthroscopic meniscectomy for degenerative meniscal tears reduces knee pain but is not cost-effective in a routine health care setting: A multi-center longitudinal observational study using data from the osteoarthritis initiative. Osteoarthr. Cartil..

[15] Bergkvist D., Dahlberg L.E., Bloch J., Neuman P., Zhou C., Englund M. (2020). Osteoarthritis and Cartilage Open Sick leave before and after arthroscopic partial meniscectomy due to traumatic meniscal tear. Osteoarthr Cartil Open.

[16] Socialstyrelsen (2004). https://www.socialstyrelsen.se/globalassets/sharepoint-dokument/artikelkatalog/klassifikationer-och-koder/2004-4-1_200441.pdf.

[17] Ludvigsson J.F., Andersson E., Ekbom A., Feychting M., Kim J., Olausson P.O. (2011). External review and validation of the Swedish national inpatient register. BMC Publ. Health.

[18] Paxton E.S., Stock M.V., Brophy R.H. (2011). Meniscal repair versus partial meniscectomy: a systematic review comparing reoperation rates and clinical outcomes. Arthroscopy.

[19] Tsarouhas A., Hantes M.E., Tsougias G., Dailiana Z., Malizos K.N. (2012). Tourniquet use does not affect rehabilitation, return to activities, and muscle damage after arthroscopic meniscectomy: a prospective randomized clinical study. Arthrosc. J. Arthrosc. Relat. Surg..

[20] Grøvle L., Haugen A.J., Keller A., Natvig B., Brox J.I., Grotle M. (2012). Poor agreement found between self-report and a public registry on duration of sickness absence. J. Clin. Epidemiol..

[21] Essen C., Mccallum Von S., Barenius B., Eriksson K. (2020). Acute reconstruction results in less sick-leave days and as such fewer indirect costs to the individual and society compared to delayed reconstruction for ACL injuries. Sport Traumatol. Arthrosc..

[22] Kiadaliri A.A., Englund M., Lohmander L.S., Carlsson K.S., Frobell R.B. (2016). No economic benefit of early knee reconstruction over optional delayed reconstruction for ACL tears: registry enriched randomised controlled trial data. Br. J. Sports Med..

[23] Skou S.T., Hölmich P., Lind M., Lind H.P., Jensen C., Thorlund J.B. (2022). Early surgery or exercise and education for meniscal tears in young adults. NEJM Evid.

[24] Persson F., Turkiewicz A., Bergkvist D., Neuman P., Englund M. (2018). The risk of symptomatic knee osteoarthritis after arthroscopic meniscus repair vs partial meniscectomy vs the general population. Osteoarthritis Cartilage.

